# 选择性XPO1抑制剂在急性髓系白血病治疗中的研究进展

**DOI:** 10.3760/cma.j.issn.0253-2727.2023.09.017

**Published:** 2023-09

**Authors:** 星 童, 莹 张, 佳 陈, 德沛 吴

**Affiliations:** 1 苏州大学附属第一医院病理科，苏州 215006 Department of Pathology, the First Affiliated Hospital of Soochow University, Suzhou 215006, China; 2 苏州大学附属第一医院血液科，苏州 215006 Department of Hematology, the First Affiliated Hospital of Soochow Universityy, Suzhou 215006, China

核输出蛋白1（exportin 1, XPO1）是细胞中最关键也是研究最广泛的核质转运蛋白，既往研究证实XPO1在多种血液肿瘤和实体瘤中均有过表达，并且与疾病进展、药物抵抗及预后相关[1]，提示它是一个潜在的肿瘤治疗靶点。XPO1抑制剂通过靶向抑制核输出，发挥多途径的抗肿瘤作用，且与多种药物发挥协同抗肿瘤作用，在急性髓系白血病（AML）靶向治疗肿瘤中的探索是近年来的研究热点。目前XPO1的选择性抑制剂塞利尼索已被批准用于复发难治性多发性骨髓瘤（MM）和弥漫大B细胞淋巴瘤（DLBCL）的治疗。在AML领域也有多项基础及临床研究的数据显示，塞利尼索单药或联合治疗均表现出良好的抗白血病效果，或将成为AML治疗的新选择。本文就塞利尼索在AML领域的研究进展作一综述，分析可进一步探索的潜在治疗组合和研究方向。

一、XPO1靶向药物在AML中的作用

XPO1又称为染色体区域稳定蛋白1（CRM1），负责将肿瘤抑制蛋白（tumour suppressor proteins，TSP）运输出核，TSP包括p53、p21、p27、p73、核磷素-1（NPM1）、FOXO3A、拓扑异构酶Ⅱ等[2]。上述蛋白的异常出核，导致蛋白功能失活，药物耐药增加，肿瘤细胞异常增殖分化[3]–[4]。Kojima等[5]在511例初治AML患者中检测XPO1蛋白的表达水平，且变量分析显示XPO1高表达是影响总生存（OS）的独立预测因子，与细胞遗传学高危和较短的生存期相关。

核输出蛋白抑制剂的发展较快，最初为leptomycin B、anguinomycins和ratjadones等药物的初步尝试，因缺乏选择性或不可逆结合导致出现严重的全身毒性而未能进入临床[6]–[7]。此后研究人员开发出一类具有高度选择性、可逆性结合的小分子口服核输出蛋白抑制剂化合物（SINE），可与XPO1的富含亮氨酸的核输出信号（NES）结合区中Cys528位点共价结合，缓慢、可逆地抑制XPO1的出核功能，恢复TSP的活性，发挥促凋亡和抗肿瘤作用，同时允许一定程度的核输出以保障正常细胞的存活及功能[8]–[9]。研究显示XPO1抑制剂可对AML细胞表现出显著杀伤活性，且不影响正常造血干细胞[10]和祖细胞[11]。实验研究显示，在AML中XPO1抑制剂可针对下列关键TSP发挥作用。

1. TP53：TP53是血液肿瘤中最常见的突变基因之一，由MDM2（一种E3泛素连接酶）调节。TP53突变与AML治疗的耐药和不良预后相关。XPO1抑制剂可诱导p53核保留，协同MDM2抑制剂阻滞p53泛素化降解，共同作用可最大限度激活p53介导的细胞凋亡[5]。

2. NPM1：超过30％的AML患者中存在NPM1突变，常与其他AML致病基因突变共存。NPM1突变导致核定位信号（NLS）替换为NES，从而转运出核，上调HOX/Meis1基因表达，维持白血病状态。XPO1抑制剂诱导其细胞，降低HOX/Meis1表达，促进AML细胞分化[5],[12]。

3. BCL2、MCL1：基础研究显示，BCL2抑制剂维奈克拉选择性作用于BCL2时，MCL1代偿性上调会导致其耐药，这在复发/难治性的AML（R/R AML）患者中尤其明显。XPO1抑制剂抑制eIF4E（真核起始因子4E）介导的BCL2和MCL1 mRNA出核翻译过程，同时下调BCL2和MCL1蛋白表达，诱导AML细胞凋亡[13]–[14]。

4. FLT3：FLT3-ITD突变与预后不良相关，尽管FLT3抑制剂有一定疗效，但FLT3受体基因突变可降低FLT3抑制剂的结合力或引起下游信号通路代偿性活化，导致耐药。研究显示，与野生型FLT3相比，FLT3突变的AML细胞对XPO1抑制剂更敏感，靶向XPO1和FLT3双重抑制，可产生协同作用，显著延长AML小鼠生存期[15]。

此外，还有研究显示，XPO1抑制剂可下调c-myc基因依赖的DNA修复基因（Rad51和Chk1）的表达水平，抑制肿瘤细胞的同源重组修复，并促进Topo Ⅱa核定位，恢复Topo Ⅱa抑制剂的药物敏感性[16]。而Ranganathan等[17]的研究也发现，地西他滨序贯XPO1抑制剂处理AML细胞，可通过上调CDKN1A和FOXO3A表达，增强抗白血病活性，相比单用塞利尼索显著延长AML小鼠生存期。上述临床前研究为靶向XPO1治疗在AML领域的临床研究探索提供了理论支持依据。塞利尼索作为唯一获批的选择性XPO1抑制剂，目前在AML领域进行了较广泛的早期临床研究。

二、XPO1抑制剂塞利尼索治疗AML的临床研究（表1）

1. 塞利尼索单药治疗AML：一项剂量递增Ⅰ期临床试验研究纳入了95例R/R AML患者，81例可评价患者中14例达到客观缓解，包括5例完全缓解（complete remission, CR）和2例不完全缓解（incomplete recovery, CRi），中位无进展生存（PFS）期5个月。塞利尼索单药治疗R/R AML研究中的临床试验Ⅱ期推荐剂量（RP2D）为60 mg，每周给药两次，对于无法耐受强化疗的老年患者，发生的非血液学不良反应大多为1～2级，包括低钠血症、疲劳、肺炎以及厌食、恶心、腹泻等胃肠道相关反应，未见其他脏器毒性，提示塞利尼索单药治疗老年R/R AML患者耐受性良好[18]。另一项Ⅱ期临床试验入组了60岁以上不能耐受高强度化疗或者骨髓移植的R/R AML患者，对照组为研究者选择性治疗，包括输血、抗感染、生长因子、羟基脲或低甲基化药物（阿扎胞苷或地西他滨）或小剂量阿糖胞苷化疗等，塞利尼索单药治疗显示出更高的CR/CRi率（11.9％对3.5％），研究结果还显示，5种蛋白质（PKIA、ZDBF2、BCL11B、FHIT和CAMK4）活性可能与塞利尼索疗效相关[19]。除此以外，一项Ⅰ期研究探索了塞利尼索在12例异基因造血干细胞移植后患者的单药维持疗效，中位维持时间达224 d，虽有7例发生急性移植物抗宿主病（aGVHD），但评估可能与塞利尼索无关。该研究结果显示，对于高危AML和骨髓增生异常综合征（MDS）患者，移植后60～100 d给予塞利尼索单药每周60 mg长期维持治疗是安全可行的，值得进一步研究[20]。

**表1:** 塞利尼索（X）治疗急性髓系白血病（AML）患者的临床研究汇总

文献	研究	例数	患者特征	研究方案
Garzon等[18]	Ⅰ期	95	R/R AML	X单药6个剂量阶梯（16.8~70 mg/m^2^），21 d或28 d为1周期
Sweet等[19]	Ⅱ期	118对57	≥60岁R/R AML	X单药对医师选择
Cooperrider等[20]	Ⅰ期	12	AML和MDS移植后	X单药（60~80 mg QW）维持12个疗程
Fiedler等[21]	Ⅱ期	42	R/R AML	X（40 mg/m^2^和60 mg BIW）联合IDA（10 mg/m^2^，第1、3、5天）和Ara-C（100 mg/m^2^，第1~7天）
Martínez Sánchez等[22]	Ⅰ期	14	R/R AML	X（60~100 mg BIW）联合FLAG-IDA
Wang等[23]	Ⅰ期	20	新诊断或R/R AML	X（60 mg和80 mg BIW）联合MA
Sweet等[24]	Ⅰ期	21	新诊断高危AML	X（60~80 mg BIW）联合DNR（60 mg/m^2^第1~3天）和Ara-C（100 mg/m^2^第1~7天）
Timothy等[25]	Ⅱ期	21对7	新诊断≥60岁AML	X（60~80 mg BIW）联合DNR（60 mg/m^2^第1~3天）和Ara-C（100 mg/m^2^第1~7天）对DA方案
Janssen等[26]	Ⅱ期	51对51	新诊断≥66岁AML	X联合DNR（60 mg/m^2^第1~3天）和Ara-C（200 mg/m^2^第1~7天）对DA方案
Bhatnagar等[27]	Ⅰ期	25	新确诊老年或R/R AML	地西他滨序贯X（23~55 mg/m^2^ BIW）
Daver等[28]	Ⅰ期	14	R/R FLT3突变AML	X联合索拉非尼

文献	X剂量或RP2D	CR/CRi［例（%）］	PFS（月）	OS（月）	移植率（%）	试验登记号
Garzon等[18]	60 mg BIW，4周/疗程	7/81（9%）	1.7	2.7	–	NCT01607892
Sweet等[19]	60 mg BIW，4周/疗程	11.9%对3.5%	–	3.2对5.6	–	NCT02088541
Cooperrider等[20]	60 mg QW，4周/疗程	9/12（75%）	2年PFS率50%	–	–	NCT02485535
Fiedler等[21]	60 mg BIW，用3周停1周	20/42（47.6%）	–	8.2	35.7	NCT02249091
Martínez Sánchez等[22]	100 mg QW，用3周停1周	5/12（42%）RP2D组66.7%	mEFS=0.8	5.1，RP2D组13.6	33	NCT03661515
Wang等[23]	80 mg BIW，用2周停2周	13/20（65%），其中R/R AML 38%，新诊断AML 83%	1年PFS率68%	1年OS率69%	57	NCT02573363
Sweet等[24]	80 mg BIW，用3周停1周	10/19（53%）	–	10.3	31.6	NCT02403310
Timothy等[25]	60 mg BIW，用3周停1周	18/21（85%）对3/7（43%）	18.6对3.6	28.0对8.8	–	–
Janssen等[26]	60 mg BIW，4周连用	59%对80%	18个月EFS率26%对45%	18个月OS率33%对58%	–	NL5748（NTR5902）
Bhatnagar等[27]	0 mg BIW，用2周停2周	8/25（32%），其中R/R AML 20%，新诊断AML 80%	5.9	5.9	–	NCT02093403
Daver等[28]	60 mg BIW，4周连用	4/11（28.6%）	–	3.5	–	–

**注** RP2D：临床试验Ⅱ期推荐剂量；R/R：复发/难治；MDS：骨髓增生异常综合征；IDA：伊达比星；Ara-C：阿糖胞苷；MA：米托蒽醌+阿糖胞苷；FLAG：氟达拉滨+阿糖胞苷+G-CSF；DNR：柔红霉素；BIW：每周两次；QW：每周1次；CR：完全缓解；CRi：不完全缓解；PFS：无进展生存；EFS：无事件生存；OS：总生存；–：无数据

2. 塞利尼索联合用药治疗AML：塞利尼索联合用药可能较单药疗效更好，与化疗联合治疗R/R AML已有一系列Ⅰ、Ⅱ期临床研究。在一项Ⅱ期临床试验中，塞利尼所联合阿糖胞苷和伊达比星治疗42例R/R AML患者，20例患者获得了CR/CRi，为骨髓移植提供机会，并且低剂量塞利尼索联合化疗在复发/难治患者中也显示了较好的耐受性[21]。同样，在另一项Ⅰ期临床试验中，塞利尼索联合FLAG-ida方案（高剂量阿糖胞苷及氟达拉滨，加用G-CSF，联合伊达比星）治疗年轻的R/R AML患者，14例患者中有3例（21.4％）发生致命的不良事件，12例患者中有5例（42％）发生应答，4例达CR，1例达CRi，4例（33％）随后接受异基因造血干细胞移植。中位OS期和无事件生存期分别为6.0（0.9～19.3）个月和1.1（0.7～19.3）个月。RP2D 100 g/周剂量组患者应答率更高，为66.7％。其中，FLT3和NPM1突变患者各有5例，分别有4例和5例获得缓解[22]。

Wang等[23]采用塞利尼索联合大剂量阿糖胞苷和米托蒽醌治疗20例新诊断或R/R AML患者，其中3例塞利尼索使用剂量为35 mg/m^2^，17例为50 mg/m^2^，均未见剂量限制性毒性。20例患者中10例CR，3例CRi，1例部分缓解（PR），6例疾病进展，总反应率为70％。在美国癌症研究协会的一项Ⅰ期临床试验中，对21例初治高危AML患者使用塞利尼索联合柔红霉素和阿糖胞苷（7+3方案）进行治疗，19例可评估疗效的患者中有8例（53％）达CR/CRi，中位OS期为10.3个月，其中6例存活患者中位OS期超过28个月，且诱导期间均未出现剂量限制性毒性。这些临床研究表明联合方案可为高危患者带来一定的生存获益，同时说明7+3方案联用塞利尼索80 mg是一个安全剂量[24]。老年初治AML患者中已有2项随机对照Ⅱ期研究报道，但结果存在差异。Timothy等[25]的研究纳入了≥60岁的初治AML患者，尽管塞利尼索组患者ASXL1突变率更高（24％对0），但CR/CRi更高（85％对43％），且中位PFS期均有延长趋势。而Janssen等[26]的研究纳入了≥66岁初治AML患者，塞利尼索联合DA方案组的3级以上感染率（55％对37％）和早期死亡率更高（18％对8％）,大多数患者过早减量或停止治疗，CR/CRi率为59％，疗效未达预期。上述结果提示对于老年或耐受性较差的AML患者，仍需谨慎探索塞利尼索剂量以平衡耐受性和疗效。

包含塞利尼索的非化疗方案也在临床探索中。与去甲基化药物地西他滨联合治疗AML的Ⅰ期研究结果显示，在25例患者中，R/R AML患者20例，不能耐受化疗的老年（大于60岁）患者5例，总反应率为40％，地西他滨序贯使用塞利尼索60 mg每周两次，连用两周可以改善该方案耐受性，提示塞利尼索与地西他滨联合治疗值得在高风险AML患者中进一步尝试[27]。与FLT3抑制剂索拉非尼[28]、Bcl-2抑制剂维奈克拉（NCT04898894）联合的研究正在进行中，用药数据同样值得关注[29]。

从目前已发表的数据来看，针对不同耐受程度AML患者以及与不同强度药物方案组合时，塞利尼索的给药剂量（剂量范围100 mg每周1次，60～80 mg每周2次）和用药频率（3周停1周或2周停2周）不尽相同，每个疗程总剂量240～320 mg，需至少停药1周以确保骨髓抑制得到恢复。除了以上已发表的数据，还有一些正在开展的临床试验，详见表2。

**表2:** 塞利尼索（X）治疗急性髓系白血病（AML）患者的正在探索的临床研究汇总

序号	患者	例数	方案	剂量	试验登记号
1	新诊断AML	58	X+VA	60 mg QW用3周停1周	NCT05736965
2	第14天MRD阳性的新诊断AML	58	X+VA	60 mg QW用2周	NCT05736978
3	R/R AML	20	X+HAAG±HMA	60 mg BIW用2周停2周	NCT05805072
4	R/R AML	50	X+HAD或X+CAG	60 mg BIW用2周停2周	NCT05726110
5	≤30岁 R/R AML	42	X+维奈克拉+化疗	–	NCT04898894

**注** R/R：复发/难治；MRD：微小残留病；VA：维奈克拉联合阿扎胞苷；BIW：每周两次；QW：每周1次；–：无数据

三、塞利尼索耐药的相关机制

尽管塞利尼索在治疗多种肿瘤时展现出良好的治疗效果，但是仍有部分患者对塞利尼索治疗无反应或在治疗后出现复发。目前的研究表明，塞利尼索耐药可能与多种机制相关。其中，肿瘤细胞在长期治疗后可能会逐渐适应塞利尼索的作用，通过上调其他代替核输出途径，降低对XPO1的依赖，从而导致塞利尼索耐药[30]。此外，一些研究表明，塞利尼索的耐药性可能与一些信号通路的活化有关，如NF-κB信号通路的激活与塞利尼索的耐药密切相关[31]。另外，AKT-FOXO3信号通路激活也被证实是参与塞利尼索耐药的关键机制[32]。还有研究表明，XPO1基因的突变可能与塞利尼索的耐药性有关[33]。

四、展望

XPO1介导的肿瘤抑制蛋白和致癌因子mRNA过度转运出核，在白血病的进展以及药物耐药中起着关键作用。塞利尼索的安全性和联合用药的疗效已获得初步证实，还需要进一步研究以探索最佳用药方案，明确和拓展适应人群。
